# Phosphorylation of Endothelin-Converting Enzyme-1c at Serines 18 and 20 by CK2 Promotes Aggressiveness Traits in Colorectal Cancer Cells

**DOI:** 10.3389/fonc.2020.01004

**Published:** 2020-07-30

**Authors:** Pablo Pérez-Moreno, Camila Quezada-Meza, Cristopher Chavez-Almarza, Ignacio Niechi, Eduardo Silva-Pavez, César Trigo-Hidalgo, Francisco Aguayo, Lilian Jara, Albano Cáceres-Verschae, Manuel Varas-Godoy, Víctor M. Díaz, Antonio García de Herreros, Verónica A. Burzio, Julio C. Tapia

**Affiliations:** ^1^Programa de Biología Celular y Molecular, Facultad de Medicina, ICBM, Universidad de Chile, Santiago, Chile; ^2^Facultad de Ciencias, Instituto de Bioquímica y Microbiología, Universidad Austral de Chile, Valdivia, Chile; ^3^Center for Integrative Biology, Faculty of Sciences, Universidad Mayor, Santiago, Chile; ^4^Programa de Virología, Facultad de Medicina, ICBM, Universidad de Chile, Santiago, Chile; ^5^Programa de Genética, Facultad de Medicina, ICBM, Universidad de Chile, Santiago, Chile; ^6^Centro de Biología Celular y Biomedicina, Facultad de Medicina y Ciencia, Universidad San Sebastián, Santiago, Chile; ^7^Unidad Asociada CSIC, Programa de Recerca en Cáncer, Departament de Ciéncies Experimentals i de la Salut, Institut Hospital del Mar d'Investigacions Médiques, Universitat Pompeu Fabra, Barcelona, Spain; ^8^Facultad de Ciencias de la Vida, Universidad Andrés Bello, Fundación Ciencia & Vida, Andes Biotechnologies SpA, Santiago, Chile

**Keywords:** phosphorylation, protein stability, aggressiveness, biomarker, cancer prognosis, pharmacological inhibitor

## Abstract

Endothelin-converting enzyme-1 (ECE1) activates the endothelin-1 peptide, which upregulates pathways that are related to diverse hallmarks of cancer. ECE1 is expressed as four isoforms differing in their N-terminal domains. Protein kinase CK2 phosphorylates the N-terminus of isoform ECE1c, enhancing its stability and promoting invasiveness of colorectal cancer cells. However, the specific residues in ECE1c that are phosphorylated by CK2 and how this phosphorylation promotes invasiveness was unknown. Here we demonstrate that Ser-18 and Ser-20 are the *bona fide* residues phosphorylated by CK2 in ECE1c. Thus, biphospho-mimetic ECE1c^DD^ and biphospho-resistant ECE1c^AA^ mutants were constructed and stably expressed in different colorectal cancer cells through lentiviral transduction. Biphospho-mimetic ECE1c^DD^ displayed the highest stability in cells, even in the presence of the specific CK2 inhibitor silmitasertib. Concordantly, ECE1c^DD^-expressing cells showed enhanced hallmarks of cancer, such as proliferation, migration, invasiveness, and self-renewal capacities. Conversely, cells expressing the less-stable biphospho-resistant ECE1c^AA^ showed a reduction in these features, but also displayed an important sensitization to 5-fluorouracil, an antineoplastic agent traditionally used as therapy in colorectal cancer patients. Altogether, these findings suggest that phosphorylation of ECE1c at Ser-18 and Ser-20 by CK2 promotes aggressiveness in colorectal cancer cells. Therefore, phospho-ECE1c may constitute a novel biomarker of poor prognosis and CK2 inhibition may be envisioned as a potential therapy for colorectal cancer patients.

## Introduction

The Endothelin-1 (ET-1) axis is involved in several cancers by promoting tumor development and progression. ET-1 is a peptide that, once activated, binds to its cognate GPCR receptor, ET_A_R (1). ET-1 activation involves processing of its precursor big-ET-1, which is catalyzed by the endothelin-converting enzyme-1 (ECE1). The ECE1 family comprises four isoforms that only differ in a small sequence at their N-terminal cytoplasmic ends (2). ECE1 overexpression has been observed to increase invasiveness of ovarian cancer cells, however, the identity of the isoform(s) responsible for this effect is unclear (3). Also, overexpression or silencing of ECE1c in prostate cancer cells was able to either increase or reduce invasiveness, respectively (4, 5). Similar findings were reported when the ECE1c isoform was overexpressed in colorectal cancer (CRC) cells, which showed significantly enhanced migration and invasiveness (6). Thus, recently ECE1c has been suggested as an emerging potential target due its role in promoting aggressiveness of several cancers (7).

Activity, localization and sorting of ECE1 have been suggested to be regulated by phosphorylation at its N-terminal end by several protein kinases, including MAPK and PKC in non-tumor cells (2, 8, 9). Also, protein kinase CK2 has been described to potentially phosphorylate ECE1c at its N-terminus (6), although the residues that are readily phosphorylated and how this post-translational modification leads to enhanced invasiveness in CRC cells remained yet unknown. This Ser/Thr kinase is elevated in a wide variety of tumors, including CRC, which has been associated to increased proliferation and survival (10–12). CK2 was identified as a metastasis-associated gene in a proteomic study using different CRC cell lines (13–15) and its levels correlated with poor CRC patient prognosis (16, 17). In fact, inhibition of CK2 with silmitasertib in CRC cells promoted early methuosis-like cell death, thereby decreasing *in vitro* tumorigenesis at later treatment times (18), although this kind of death has been also ascribed to a CK2-independent mechanism (19). At the cellular level, CK2 has been proposed to promote survival of CRC and breast cancer cells by activating the Wnt/β-catenin signaling pathway through AKT phosphorylation (20, 21), which increases the expression of many β-catenin targets, including both the inhibitor of apoptosis survivin (22) and cyclooxygenase-2 (23). Indeed, ET-1 is a β-catenin target whose expression has been found increased in several cancers (24). CK2 also phosphorylates many substrates involved in the regulation of several signaling pathways (10). In colon and breast cancer cells, CK2 has been shown to promote activation of PI3K/Akt/mTOR, NF-κB and JAK/STAT signaling pathways related to the acquisition of malignant traits, such as exacerbated proliferation and death resistance (25–27).

Here, we have precisely identified Ser-18 and Ser-20 as the *bona fide* residues phosphorylated by CK2 in ECE1c, which enhances its protein stability. Moreover, by constructing biphospho-mimetic and biphospho-resistant mutants, the role of CK2-mediated stability of ECE1c in modulating some hallmarks of cancer was assessed in CRC cells. The findings presented in this work show that phosphorylation of ECE1c at Ser-18 and Ser-20 by CK2 promotes aggressiveness of CRC cells, which allows to suggest phospho-ECE1c as a novel biomarker of poor prognosis in CRC patients.

## Materials and Methods

### Cell Culture and Stable Cloning

DLD-1 (ATCC® CCL-221™) colorectal cancer cells were kindly provided by Dr. J. Silvio Gutkind (Department of Pharmacology, UC San Diego Moores Cancer Center, CA, US). HT-29 (ATCC® HTB-38™) colorectal cancer cells were kindly provided by Dr. Andrew F. Quest (Faculty of Medicine, University of Chile). Cells were expanded in RPMI 1640 medium supplemented with 10% FBS and antibiotics (10,000 U/ml penicillin, 10 μg/ml streptomycin) at 37°C and 5% CO_2_, followed by storage in liquid nitrogen at −190°C. Once a year, an aliquot was thawed, expanded and stored at −80°C. For experiments, one aliquot was thawed and grown as above. Cells were used for experiments within one calendar year and eliminated at the 15th passage as requested by our local biosecurity committee. Mycoplasma was tested every month with the EZ-PCR Mycoplasma Test kit (Biological Industries, Beit Haemek, Israel). For stable cell cloning, a pLVX-IRES-mCherry bicistronic lentiviral vector (Clontech) was used. Lentiviral vector production was carried out using the Lenti-X™ 293T cell line (Clontech) by transfection of a 2nd generation lentiviral system with a calcium phosphate protocol (28), under biosecurity conditions. Cells were transduced with the lentiviral particles encoding each Flag/myc-tagged ECE1c mutant and expanded for 1 week. The mCherry reporter on the lentiviral vector was used to sort the transduced positive brightest cells to almost 100% purity on a FACSAria Fusion cell sorter (Becton Dickinson). The sorted pooled cells were expanded and purity of the mCherry-positive cells was evaluated by flow cytometry. Purity of the sorted cells expressing the mCherry reporter was checked after several passages, corroborating the stability of expression of the lentiviral transgene, including the Flag-ECE1c mutants.

### Mass Spectrometry

Recombinant purified GST-NT-ECE1c protein [Niechi et al. (6)] was used as substrate for commercial human CK2 (Biaffin GmbH & Co, KG) in the presence of ATP (Sigma), following manufacturer's instructions. Samples were treated with 1 U/μl trypsin (Gibco) and peptides were separated by liquid chromatography (Waters 2,695 Separation Module) coupled to an electrospray positive ionization mass spectrometer (ESI+/Waters Quattro Micro) located at CRG/UPF Proteomics Unit, Barcelona, Spain. The sequences obtained were analyzed against a human protein database (sp_human_2015_04_VDAH) using the Mascot software v1.4 (Matrix Science).

### Western Blot

Cells were harvested, lysed with RIPA and 30 μg of total protein were separated on a 10% SDS-PAGE gel. Gels were transferred to nitrocellulose membranes (Macherey-Nagel) and probed with primary antibodies targeting Flag (mouse monoclonal; 1:4000; OriGene), β-actin (goat polyclonal; 1:2000; Santa Cruz Biotechnology), P-FAK (rabbit monoclonal; 1:1000; Cell Signaling), FAK (mouse monoclonal; 1:1000; Santa Cruz Biotechnology), cyclin D1 (rabbit monoclonal; 1:1000; Cell Signaling) and survivin (rabbit polyclonal; 1:2000; R&D Systems). After incubation with an HRP-conjugated mouse anti-IgG (Santa Cruz, 1:2000), membranes were revealed using EZ-ECL kit (Biological Industries, Haemek, Israel) and analyzed on a ChemiDoc Imaging System (Bio-Rad).

### Immunofluorescence and Confocal Microscopy

Cells overexpressing Flag-tagged ECE1c^WT^, ECE1c^AA^, or ECE1c^DD^ proteins were grown on glass coverslips. After 48 h, cells were washed with PBS, fixed in 3.7% p-formaldehyde for 15 min, permeabilized for 10 min at RT in 0.2% Triton X-100/PBS, blocked with 3% BSA/PBS for 30 min at RT and incubated with mouse anti-Flag antibody in 1% BSA/PBS (1:1000, OriGene) for 1 h at RT. Incubation with secondary antibody (1:500) was performed in 1% BSA/PBS for 45 min at RT in the dark. Nuclei were stained with DAPI for 10 min (Thermo Fisher Scientific). Images were acquired with a 60x objective lens under a Nikon C2 plus confocal microscope. Confocal 8-bit RGB images were processed with ImageJ software (National Institutes of Health). Representative cells are shown in all figures at the same magnification.

### Protein Stability

Cells (1.2 × 10^6^) were seeded into p60 plates and cultured for 16 h at 37°C and 5% CO_2_ in complete medium and then incubated with 20 μg/ml cycloheximide (CHX) in the absence or presence of 25 μM silmitasertib. Cells were harvested after 0, 3 and 6 h of treatment and cell extracts were analyzed by Western blot, using an anti-Flag antibody.

### Flow Cytometry

For proliferation analysis, cells were detached by trypsinization, washed and left in PBS to a density of 10^6^ cells/ml. CFSE (eBioscience) was added to a final concentration of 5 μM, mixed for 10 s and incubated for 10 min. One volume of FBS was added, followed by complete medium and cells were then pelleted, washed, seeded into p60 plates, and incubated for the indicated times. For cell cycle analysis, cells were incubated with 25 μM 5-fluorouracil (5-FU) for the indicated times and then centrifuged at 1,000 × *g* for 5 min at RT, washed in 1 ml cold PBS, suspended in 1 ml staining solution (0.1% Triton X-100, 50 μg/ml PI and 200 μg/ml RNAse) and incubated for 30 min at 37°C in the dark. All assays were analyzed on a Becton-Dickinson LSR Fortessa X-20 flow cytometer and the FACSDiva 8.02 software (San Jose, CA) at the MED.UCHILE-FACS Facility, Facultad de Medicina, Universidad de Chile.

### RT-qPCR

Total RNA was extracted from cells using the EZNA Total RNA Kit I (Omega bio-tek, Giorgia USA) and treated with DNase (DNA-free Kit Ambion, Life Technologies). RNA concentration was measured with NanoQuant Infinite M200 pro spectrophotometer (Tecan). Reverse transcription was performed using the AffinityScript QPCR cDNA Synthesis Kit (Agilent Technologies, TX). Quantitative real-time PCR was performed in a StepOne real-time PCR system (Applied Biosystems) with SYBR Green PCR master mix (Thermo-Fisher Scientific, Vilnius, Lithuania). Determinations were performed in triplicate (40 cycles) and the relative abundance of each mRNA was determined by the 2^−ΔΔct^ method and normalized to GAPDH. The 95% confidence interval was determined to indicate variability of the mean ratios for each experiment. Primers used were: Cyclin-D1 FW 5′- GGATGCTGGAGGTCTGCGA-3′, RV 5′- AGAGGCCACGAACATGCAAG-3′; Survivin Fw 5′-CTGCGCAGCCCTTTCTCAAGGA-3'; Rv 5'-GCAACCGGACGAATGCTTTT-3'; GAPDH FW 5'-GAGTCAACGGATTTGGTCGT-3', RV 5'-GACAAGCTTCCCGTTCTCAG-3'.

### Migration

Migration was assessed in Boyden chambers (6.5 mm diameter, 8 μm pore) according to manufacturer's instructions (Transwell Costar). Briefly, the bottom of each insert was coated with 0.5 ml 2 μg/ml fibronectin. Cells (4 × 10^4^) were suspended in serum-free medium and seeded onto the top of each chamber insert. Medium supplemented with 10% FBS was added to the bottom chamber. After 8 h, inserts were removed, washed and cells attached to the lower side were stained with 0.1% crystal violet in 20% methanol. Migrated cells were scored under a Nikon Eclipse TS100 inverted microscope.

### Invasion

Cells (4 × 10^4^) in RPMI medium without FBS were seeded in matrigel chambers (Corning) and deposited into 24-well plates with 500 μl of RPMI/10% FBS per well. Cells were incubated during 22 h at 37°C and 5% CO_2_. Then, chambers were stained and fixed with 0.05% crystal violet in 20% methanol for 1 h. Invasive cells were counted under a Nikon Eclipse TS100 inverted microscope.

### Colony-Formation

Cells (2.5 × 10^3^) were suspended in 0.33% soft Bacto-agar (BD Biosciences) in RPMI-1640 containing 12.5% FBS. The cell suspension was then poured into 6-well plates containing a layer of 2 ml 0.5% agar in the same media. Plates were fed twice a week with 0.5 ml RPMI-1640 supplemented with 10% FBS. After 21 days, colonies were photographed under a Nikon Eclipse TS100 inverted microscope. Finally, cells were stained using 0.005% crystal violet dissolved in 20% methanol for 1 h at RT and colonies visible to the naked eye were also photographed and documented with a Nikon D5100 camera.

### Sphere-Formation

Cells (5 × 10^4^) were seeded into agarose-coated 6-well plates (Corning) in Mammary Epithelial Growth Medium (MEGM; Lonza) supplemented with 0.5 g/ml hydrocortisone, 25 ng/ml EGF, 5 μg/ml insulin (Lonza) and 25 ng/ml bFGF (Invitrogen). After 7 days in culture, spheres over 80 μm in diameter were scored under a Nikon Eclipse TS100 microscope, using the Micrometrics SE Premium 4 software.

### Statistics

All values were expressed as mean ± SEM of at least three independent experiments. Statistics were performed using ANOVA and Bonferronni as a post-test with the Graph Pad Prism5 software. Statistical significance (*p*-value) was set at a nominal level of 0.05 or less.

## Results

### CK2 Phosphorylates ECE1c at Serines-18/20 and Enhances Its Stability

**Figure 1:**
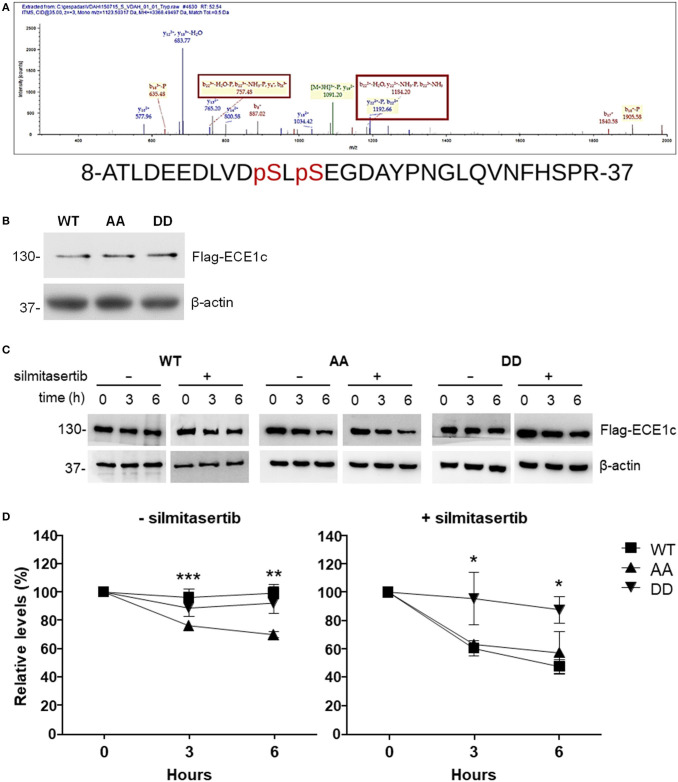
CK2 phosphorylates ECE1c on serines 18 and 20, enhancing its stability. **(A)** Phosphorylation was performed with commercial human CK2 and recombinant GST-fused N-terminus of ECE1c and ATP as substrates. Samples were analyzed by ESI^+^-MS. A representative spectrogram of one of the tryptic peptides is shown with phosphorylated serines enclosed in squares. **(B)** DLD-1 cells overexpressing Flag-tagged ECE1c^WT^, ECE1c^AA^, or ECE1c^DD^ were grown for 12 h under normal conditions and 10 μg of total lysates were analyzed by Western blot with an anti-Flag antibody using β-actin as loading control. **(C)** Cells were grown for 12 h and then incubated with 20 μg/ml cycloheximide (CHX), with or without 25 μM silmitasertib, during the indicated times. Detection of ECE1c proteins was performed as in **(B)**. Representative blots are shown. **(D)** Relative levels of Flag-ECE1c proteins from 3 independent experiments as in C were evaluated. Graphs represent mean ± SEM; *p < 0.05, **p < 0.01, and ***p < 0.001 (for AA vs. DD).

In order to identify the precise residue(s) phosphorylated by CK2, a recombinant GST-fused N-terminal end of ECE1c was used as substrate for a non-radioactive *in vitro* CK2 kinase assay. Trypsin-digested phosphorylated samples were analyzed by mass spectrometry and sequences obtained were *in silico* evaluated against a human protein database. The results showed that Ser-18 and Ser-20 are the *bona fide* residues phosphorylated by CK2 with a 100% confidence (Figure 1A).

To assess the role of phosphorylation of Ser-18 and Ser-20 in ECE1c and possible consequences in some hallmarks of cancer, we prepared clones of CRC cells overexpressing Flag-tagged biphospho-mimetic (ECE1c^DD^), biphospho-resistant (ECE1c^AA^) and wild-type (ECE1c^WT^) forms. As shown in Figure 1B, no important changes in protein levels were observed for each ECE1c form in DLD-1 colorectal cancer cells grown 12 h under normal conditions. However, treatments with the eukaryotic translational elongation inhibitor, cycloheximide (CHX), in the absence or presence of the CK2 inhibitor silmitasertib, revealed significant differences in the stability of the different forms of ECE1c (Figure 1C). In the absence of silmitasertib, the biphospho-mimetic ECE1c^DD^ mutant was as stable as the wild-type ECE1c^WT^ form and its levels only dropped to >90% after 6 h of treatment with CHX (Figure 1D-left). However, in the presence of silmitasertib, the ECE1c^DD^ mutant was by far the more stable form, while the stability of the ECE1c^WT^ significantly dropped to 60% even at 3 h of growth (Figure 1D-right). Importantly, the biphospho-resistant ECE1c^AA^ mutant displayed the lowest stability, independently of treatment with silmitasertib, with a significant reduction of its levels to 70 or 57% in the absence or presence of the CK2 inhibitor for 6 h, respectively (Figure 1D). Altogether, these results indicate that ECE1c is specifically phosphorylated by CK2 at residues Ser-18 and Ser-20, which highly enhances its protein stability.

### Absence of CK2-Mediated Phosphorylation of ECE1c Sensitizes CRC Cells to 5-FU

**Figure 2:**
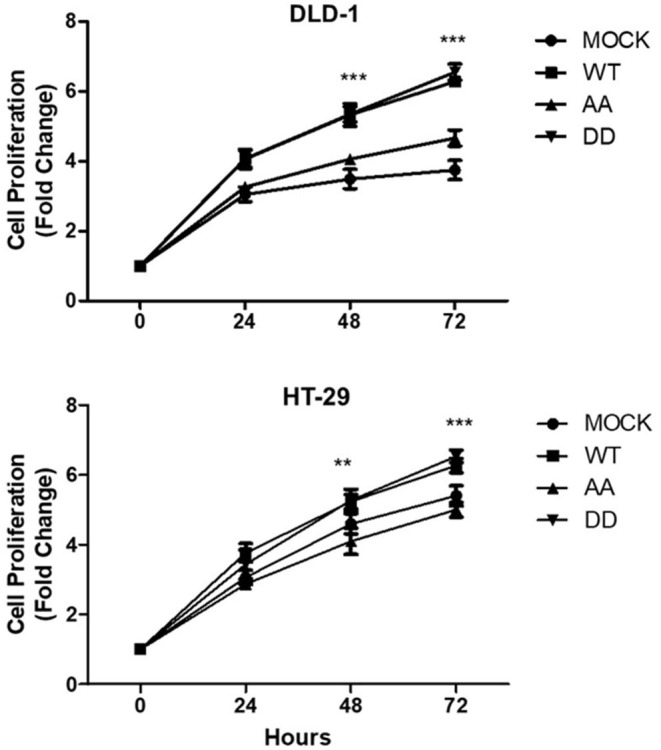
Biphospho-mimetic ECE1c^DD^ increases proliferation of CRC cells. DLD-1 (upper) and HT-29 (lower) CRC cells overexpressing Flag-tagged ECE1c^WT^, ECE1c^AA^, or ECE1c^DD^ were grown for 24, 48, and 72 h under normal conditions and proliferation was evaluated by CFSE assay and flow cytometry. Graphs represent mean ± SEM; **p < 0.01; ***p < 0.001.

To evaluate the effect of CK2-dependent phosphorylation of ECE1c on cell proliferation, we used a CFSE-based cytometric assay with stable clones of the different forms of ECE1c prepared on two broadly used CRC cell lines, such as HT-29 and DLD-1. Augmented proliferation was observed in both CRC cell lines expressing ECE1c^WT^ and ECE1c^DD^ in comparison to mock cells, which was significant from 48 h of growth. Interestingly, the lack of the two potentially phosphorylated serines in ECE1c^AA^ did not have any significant effect in proliferation in comparison to mock cells, at least after 72 h of growth in both CRC cell lines (Figure 2). Of note, a similar cell proliferation profile was observed by Trypan blue staining of DLD-1 cells (Supplementary Figure 1).

**Figure 3:**
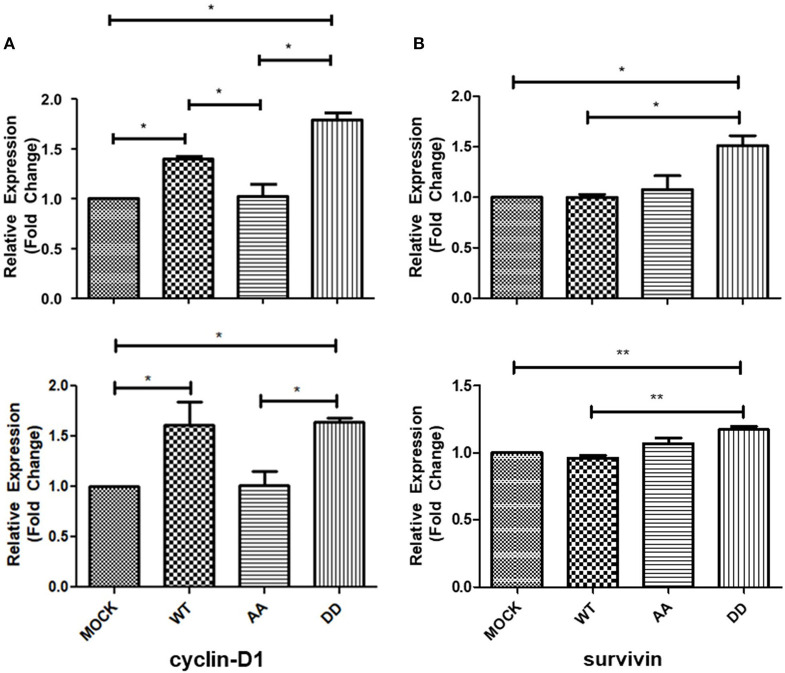
Increased mRNA levels of cyclin-D1 and survivin upon expression of ECE1c^DD^ in CRC cells. HT-29 (upper) and DLD-1 (lower) cells overexpressing Flag-tagged ECE1c^WT^, ECE1c^AA^, or ECE1c^DD^ were grown for 24 h under normal conditions and mRNA levels of cyclin-D1 **(A)** and survivin **(B)** were measured by RT-qPCR. Graphs represent mean ± SEM; *p < 0.05, **p < 0.01.

**Figure 4:**
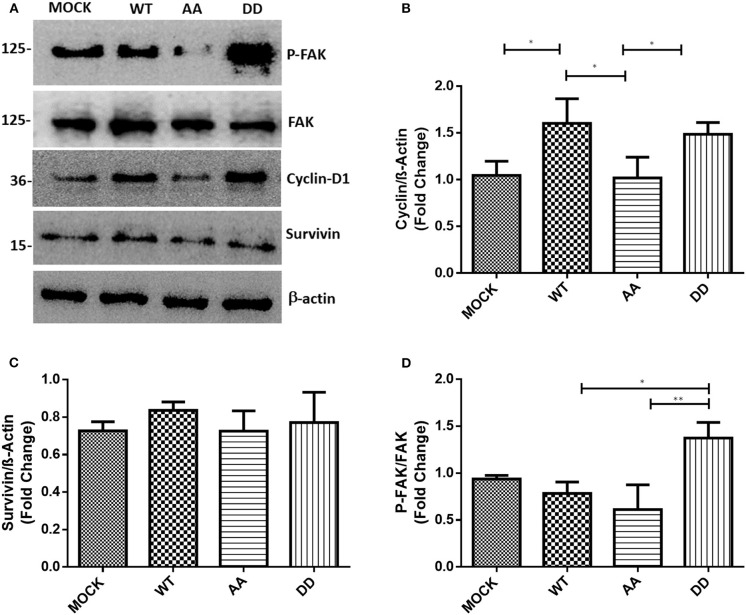
Biphospho-mimetic ECE1c^DD^ increases protein levels of cyclin-D1 and phospho-FAK. **(A)** DLD-1 cells overexpressing Flag-tagged ECE1c^WT^, ECE1c^AA^, or ECE1c^DD^ proteins were grown for 24 h under normal conditions and protein levels of phospho-FAK (P-FAK), FAK, cyclin-D1, and survivin were detected by Western blot using specific antibodies. Representative blots are shown. Relative β-actin-normalized levels of cyclin-D1 **(B)** and survivin **(C)**, and P-FAK/total FAK ratio **(D)** were quantified with ImageJ. Graphs represent mean ± SEM; *p < 0.05, **p < 0.01.

The above results suggested that biphospho-mimetic ECE1c^DD^ overexpression may positively affect cell cycle progression of these CRC cancer cells. It is well-described that cyclin-D1 regulates cell cycle transition from G1 to S phase, a process aberrantly dysregulated in human cancers (29). On the other hand, survivin regulates microtubule stability and mitotic progression (30), although its role in CRC is more strongly linked to inhibition of apoptosis in a CK2-dependent manner (22). Thus, mRNA levels of cyclin-D1 and survivin were measured in CRC cells growing for 24 h under normal conditions. RT-qPCR quantification showed an increase in cyclin-D1 and survivin mRNA levels in ECE1c^DD^-expressing cells as compared to mock cells (Figure 3). Notably, cyclin-D1 mRNA levels were significantly higher in ECE1c^DD^- and ECE1c^WT^-expressing cells (Figure 3A), while elevated survivin mRNA levels were only observed in ECE1c^DD^-expressing cells (Figure 3B). This coincided with an increased % of subG_0_ population, suggesting an enhanced progression through the G1 phase (Supplementary Table 1). Moreover, only cyclin-D1 protein levels significantly increased, in accordance with mRNA levels, in both ECE1c^DD^- and ECEc1c^WT^-expressing cells after 24 h of growth (Figures 4A,B), which was similarly observed in both DLD-1 and HT-29 (not shown) cell lines. This suggested that protein expression of survivin is probably more strongly regulated at 24 h, becoming detectable and functional at longer times and/or under exposition to a noxious compound (Figure 4C). Overall, our results allow to explain the increased progression through the G1 phase observed at 24 h of growth, followed by an augmented proliferation at longer times only in biphospho-mimetic ECE1c^DD^-expressing CRC cells.

**Figure 5:**
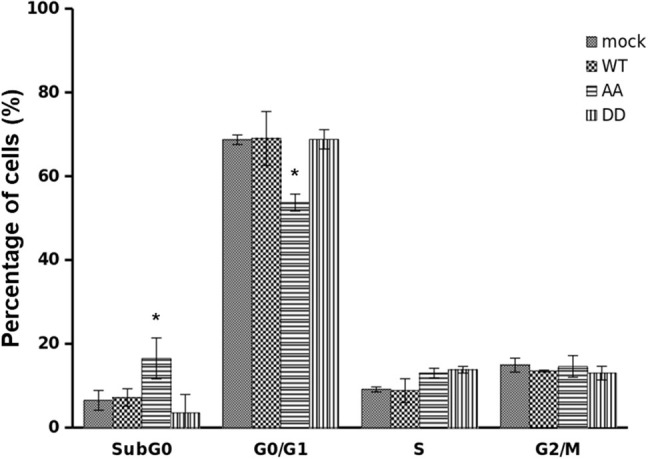
Biphospho-resistant ECE1c^AA^ expression impinges progression through G1 and sensitizes CRC cells to death by 5-fluorouracil. DLD-1 cells overexpressing Flag-tagged ECE1c^WT^, ECE1c^AA^, ECE1c^DD^ proteins, or mock-transduced cells were grown for 24 h in the presence of 25 μM 5-fluorouracil (5-FU) and cell cycle analysis was performed by flow cytometry. Graph represents mean ± SEM; *p < 0.05.

The ability of ECE1c^DD^ to enhance CRC cell proliferation at relatively longer times suggested that CK2-mediated phosphorylation of ECE1c may lead to enhanced resistance to an antineoplastic drug. Therefore, we treated our cells for 24 h with 25 μM 5-fluorouracil (5-FU), a traditional antineoplastic drug used in CRC therapy. Cytometric analysis showed no significant differences in S and G2/M populations for all cells treated with 5-FU. However, the biphospho-resistant ECE1c^AA^-expressing cells experimented a dramatical increase in subG_0_ population (Figure 5), indicating the onset of apoptosis-related death upon treatment with 5-FU, as suggested by our published results with another apoptosis-inducing drug in the same cells (18). In contrast, the biphospho-mimetic ECE1c^DD^-expressing cells displayed the lowest % of subG_0_ population. Likewise, ECE1c^AA^-expressing cells displayed the lowest % of G_0_/G_1_ population upon treatment with 5-FU, while ECE1c^DD^-expressing cells showed the highest % of G_0_/G_1_ together with ECE1c^WT^-expressing and mock cells. These findings show that blocking phosphorylation of ECE1c by CK2 both impinges progression through the G1 phase and sensitizes CRC cells to death by the drug 5-FU.

### Biphospho-Mimetic ECE1c^DD^ Promotes Migration and Invasion of CRC Cells

**Figure 6:**
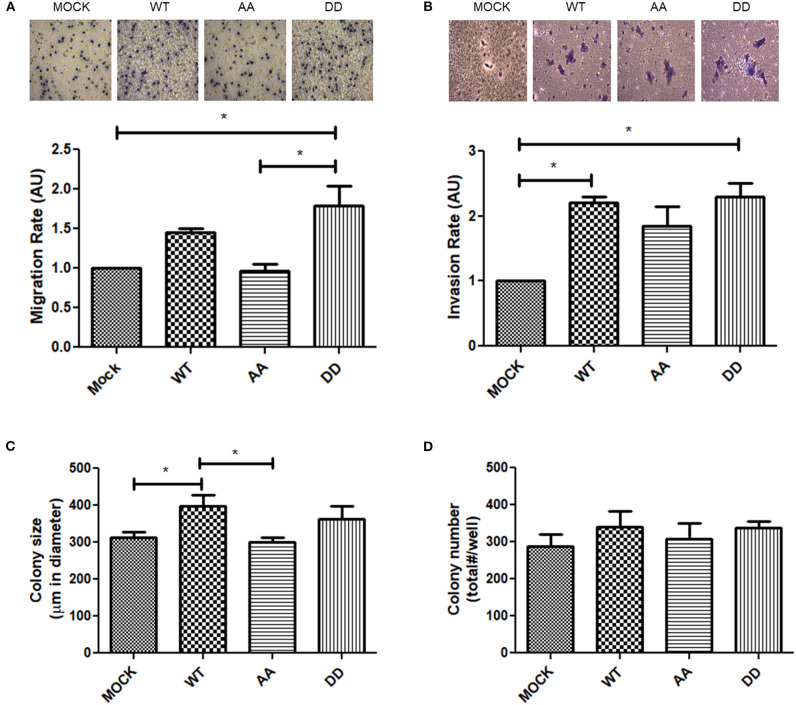
Migration, invasion and colony-formation upon expression of ECE1c proteins in CRC cells. **(A)** Migration capacity of DLD-1 cells was evaluated at 8 h of incubation in transwell assay. Representative images of crystal violet-stained cells at 40x magnification (upper panel). Relative quantification of migrated cells (lower panel). **(B)** Invasion capacity of DLD-1 cells was evaluated for 22 h by matrigel assay. Representative images of crystal violet-stained cells at 40x (upper panel). Relative quantification of migrated cells (lower panel). **(C,D)** Colony formation was evaluated for 21 days by soft-agar assay. Number **(C)** and size **(D)** of colonies was measured with ImageJ and Micrometrics SE Premium 4 software, respectively. Graphs represent mean ± SEM. *p < 0.05.

ECE1 silencing or overexpression have been described to decrease or increase phosphorylation (i.e., activation), respectively, of focal adhesion kinase (FAK) in prostate cancer cells (31). FAK is a cytoplasmic Tyr-kinase that promotes metastasis in several cancers by phosphorylating cytoskeleton proteins and remodeling the ECM via MMPs (32). Thus, only biphospho-mimetic ECE1c^DD^ expression let to an increased relative phospho-FAK levels, indicative of activation of this migration-related kinase (Figure 4D). This correlated with significantly augmented migration and invasion capabilities which were almost identical in DLD-1 (Figures 6A,B) and HT-29 (not shown) cells. Additionally, confocal microscopy analysis of subcellular localization of the Flag-ECE1c forms in DLD-1 cells showed an apparent cytoplasmic spread pattern of ECE1c^DD^ as compared to WT and AA forms, which were close to the ER/Golgi compartments (Supplementary Figure 2). Interestingly, an increased ECE1 activity in intracellular compartments has been correlated with a positive ET-1 immunoreactivity in secretory vesicles (33). Whether these apparent subcellular patterns of our ECE1c forms are linked to the differential migration and invasion capabilities observed in CRC cells and whether they correlate with big-ET-1 processing, warrants further work mainly by using different compartment markers, such as GM130 and E-cadherin, among others. Despite the above, we assessed the putative role of the CK2-mediated phosphorylation of ECE1c in *in vitro* tumorigenesis through a traditional soft-agar assay. Unexpectedly, after 21 days of growth in soft-agar, colony number and size did not differ between the biphospho-mimetic ECE1c^DD^-expressing cells and the other clones (Figures 6C,D). These results suggest that phosphorylation of residues Ser-18 and Ser-20 by CK2 may favor only metastasis-linked capabilities of CRC cells.

### Phosphorylation of ECE1c Enhances Aggressiveness of CRC Cells

**Figure 7:**
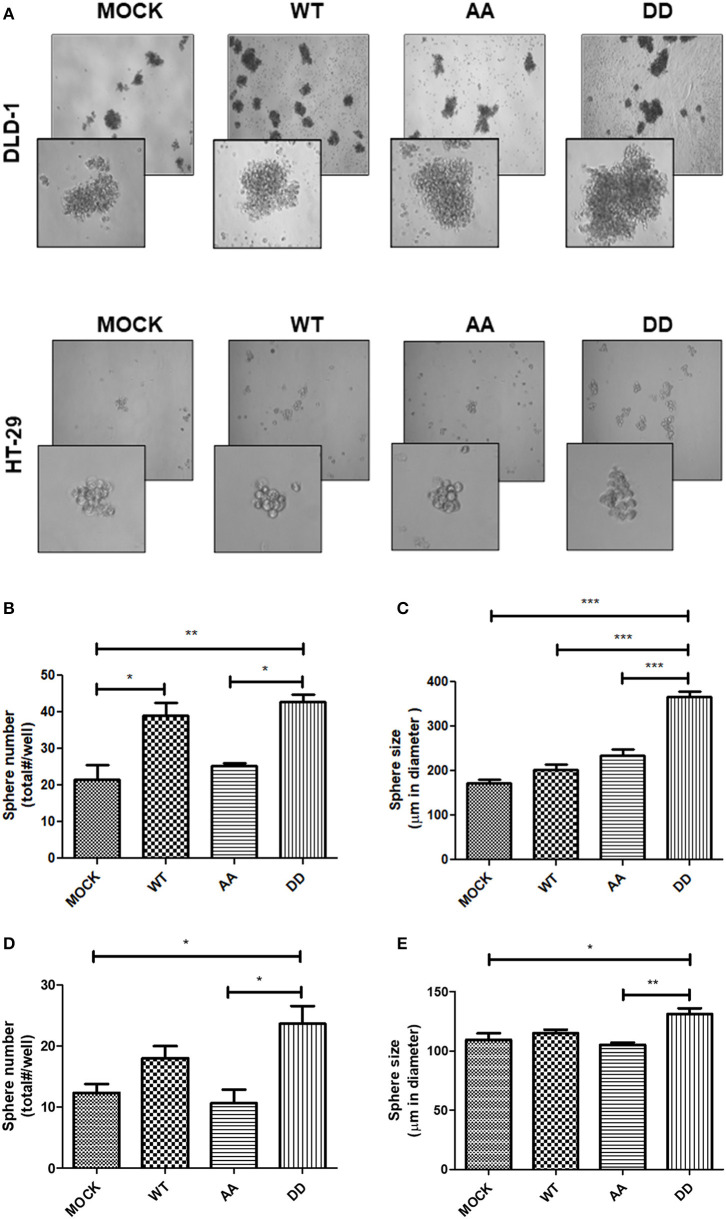
Biphospho-mimetic ECE1c expression enhances aggressiveness of CRC cells. CRC cells stably overexpressing Flag-tagged ECE1c^WT^, ECE1c^AA^, or ECE1c^DD^ were grown in MEGM in agarose-coated wells during 7 days. **(A)** Representative images were taken at 40x and 100x (insert) magnifications by phase-contrast microscopy of DLD-1 (upper) and HT-29 (lower) cells. Spheres over 80 μm in diameter formed by DLD-1 **(B)** and HT-29 **(C)** clones were counted and plotted as total spheres/well. Size of spheres formed by DLD-1 **(D)** and HT-29 **(E)** clones was measured using the Micrometrics SE Premium 4 software and plotted as sphere diameter in μm. Graphs represent mean ± SEM. *p < 0.05, **p < 0.01, ***p < 0.001.

The above findings suggested that CRC cells overexpressing either the native full-length ECE1c^WT^ or the biphospho-mimetic ECE1c^DD^ proteins may display enhanced self-renewing capability. In order to evaluate this important cancer-related hallmark, an anchorage-independent spheroid-formation assay was performed during 7 days in culture under anchorage-independent conditions (Figure 7A). As observed, a significantly higher number of spheres was obtained with both ECE1c^DD^- and ECE1c^WT^- expressing cells, compared to biphospho-resistant ECE1c^AA^-expressing and mock cells in both CRC cell lines (Figures 7B,D). Interestingly, the number of spheres displayed a very similar pattern to that of the cyclin-D1 expression, migration and invasion capabilities. In DLD-1 cells, significantly larger spheres were obtained only with ECE1c^DD^-expressing cells (Figure 7C), while in HT-29 cells spheres were significantly larger compared only with ECE1c^AA^-expressing and mock cells (Figure 7E). These findings indicate that increased ECE1c stability promoted by CK2 phosphorylation at Ser-18 and Ser-20 leads to enhanced aggressiveness of CRC cells through improved self-renewal capacity.

## Discussion

ECE1 expression occurs in normal tissues and increased levels have been detected in samples of patients with different cancers (7). ECE1 is expressed as four isoforms encoded by a single gene in which exons 1–3 suffer alternative splicing, giving rise to four distinct isoforms (ECE1a, ECE1b, ECE1c, and ECE1d), whose main difference lies in a short N-terminal cytoplasmic domain (34). The N-terminus sequence is remarkably conserved in the animal kingdom, where residues Ser-18 and Ser-20 are present in isoforms ECE1a, ECE1b and ECE1c, however, ECE1c is the most highly expressed isoform in all tissues and consequently in several cancers (35, 36). Moreover, only isoform ECE1c has been demonstrated to promote invasiveness potential in prostate (4, 5) and colorectal (6, 37) cancer cells. Thus, ECE1c has been suggested as a putative therapeutic target, due to its role in promoting cancer aggressiveness (7).

Although Ser-18 and Ser-20 are also present at the N-terminus of isoforms ECE1b and ECE1d, our current results show for the first time *in vitro* that ECE1c is phosphorylated at both serines by protein kinase CK2. In a search for the occurrence of natural mutations at Ser-18 and Ser-20, which could shed light on a role of CK2 in CRC patients, 3953 CRC samples were analyzed *in silico* through the cBioPortal software (https://www.cbioportal.org). This genomic study showed that the main mutations in CRC samples are located in the catalytic domain of ECE1c, with only two mutations occurring at the N-terminus, X18 and A40V, which are related to alternative splicing and missense, respectively. The fact that CK2 can phosphorylate ECE1c at Ser-18 and Ser-20 *in vitro* suggests a putative CK2-ECE1c interaction. Even though formation of a putative complex may be studied by immunoprecipitating ECE1c^WT^ from transfected or normal cells with an anti-CK2-phospho-substrate antibody, our findings allow to predict a positive result for that kind of analysis. In spite of this, a similar result has been reported by immunoprecipitation with an anti-pan-antibody for ECE1 from lysates of DLD-1 cells grown in the absence and presence of a CK2 inhibitor, TBB. Therein, ECE1 phosphorylation, detected with an anti-phospho-Ser/Thr/Tyr antibody, was strongly diminished in TBB-treated DLD-1 cells (6).

Serines 18 and 20 at the N-terminal end of ECE1 had been reported to be constitutively phosphorylated by CK1 in non-tumor HUVEC cells (38), however, the same residues were later shown to be phosphorylated by a MAPK (2, 9). Thus, the exact identity of the kinase responsible for the phosphorylation of these residues in a specific cellular context was an open question. Other phosphorylations at the N-terminus of ECE1c were reported to occur, for example by PKC, which increases its localization and activity at the plasma membrane (39). Besides CK1, MAPK and PKC, other phosphorylations had not been comprehensively studied. Therefore, the increased stability of ECE1c brought about by phosphorylation at Ser-18 and Ser-20, resulting in the enhancement of malignant properties *in vitro* in CRC cells is, to our knowledge, a novel and relevant finding.

Our results also show that phosphorylation of ECE1c at Ser-18 and Ser-20 leads to enhanced stability. Nevertheless, enhanced stability of the biphospho-mimetic ECE1c^DD^ mutant could be observed only after a 3 h treatment with cycloheximide, since amounts detected in transfected cells were almost identical at zero time. Although this could be explained through a compensatory transcriptional mechanism, data published by our group suggests that the enhanced stability of ECE1c by CK2-mediated phosphorylation may abrogate its proteasomal degradation (6, 37). But ECE1c is not the only protein whose stability has been found to be regulated by CK2. This kinase phosphorylates and prevents proteasomal degradation of c-Myc, which stimulates transcription of genes involved in cell cycle progression (40). Another example is β-catenin, whose phosphorylation by CK2 precludes its binding to axin, avoiding its proteasomal degradation (41). Also linking CK2 with the proteasome, this kinase phosphorylates the deubiquitinase OTUB1, modulating its nuclear deubiquitination activity and thereby stabilizing chromatin-binding proteins (42). Thus, a high activity/level of CK2, which is normally found in many cancers (43), may prevent proteasomal degradation of ECE1c as reported here, promoting several hallmarks of cancer in CRC cells and probably prostate cancer cells (5). The latter may potentially occur in many different cancer cells in which ECE1c and CK2 are physiologically relevant (7).

Besides Ser-18 and Ser-20, the N-terminus of ECE1c contains a third residue, Thr-9, which fulfills the canonical requirements as a substrate of CK2. The fact that Thr-9 was not shown to be phosphorylated by CK2 in this work does not come as a surprise. Many canonical phospho-sites that have been proposed for CK2 in different protein substrates and even tested *in vitro* by kinase assays, have never been validated *ex vivo*, that is in a cellular context (43). Thus, a putative phosphorylation *ex vivo* of Thr-9 cannot be ruled out based on our results. However, the fact that the migration and invasiveness patterns obtained elsewhere (6) and currently from our biphospho-mutants had been very similar, allows to predict a null effect of Thr-9 on the aggressiveness traits studied here.

The data presented in this work can be rationally explained by a mechanism in which stable ECE1c leads to increased ET-1 production and subsequent activation of its cognate receptor ET_A_R, following downstream signaling for malignant traits in CRC cells. Although ET-1 production was not assessed in the present work, we have already measured ET-1 at supernatants of ECE1c^WT^- and stable mutant ECE1c-expressing cells, which were elevated as expected (6, 37). However, these findings do not allow to completely ensure an ET1-mediated effect, as unpublished results from our group showed that viability of ECE1c^WT^-expressing cells was not affected by treatment with the ET_A_R antagonist, BQ-123, in comparison to mock cells, which was significantly decreased (not shown). Indeed, a non ET-1-mediated effect of ECE1 on some malignant traits has been observed in several types of cancer cells as reviewed elsewhere (7). Moreover, our findings of the enhanced sphere-forming capability of biphospho-mimetic ECE1c^DD^-expressing cells strongly suggests that an enhanced stability of ECE1c could lead to augmented aggressiveness. Therefore, in addition to the current findings, it would suggest phospho-ECE1c as a novel biomarker of poor prognosis for patients suffering this devastating disease.

## Data Availability Statement

The raw data supporting the conclusions of this article will be made available by the authors, without undue reservation, to any qualified researcher.

## Author Contributions

PP-M, CQ-M, CC-A, and IN performed MTS, western blots, RT-qPCR, adhesion, migration, and invasion assays. PP-M, ES-P, and VB performed soft-agar and spheroid-formation assays. FA and CT-H performed flow cytometry assay. IN, VD, and AG performed mass spectrometry analysis. AC-V and MV-G developed lentiviral transduction. JT and PP-M wrote the manuscript which was reviewed and approved by LJ and VB. All authors contributed to the article and approved the submitted version.

## Conflict of Interest

VB was employed by company Andes Biotechnologies SpA. The remaining authors declare that the research was conducted in the absence of any commercial or financial relationships that could be construed as a potential conflict of interest.

## Supplementary Material

Supplementary Figure 1 ECE1c^DD^ increases proliferation of CRC cells. DLD-1 cells overexpressing Flag-tagged ECE1c^WT^, ECE1c^AA^, or ECE1c^DD^ were grown for 24, 48, and 72 h under normal conditions and proliferation was evaluated by Trypan blue staining. Graph represents mean ± SEM; ^*^*p* < 0.05; ^***^*p* < 0.001.

Supplementary Figure 2 Subcellular localization of ECE1c^WT^, ECE1c^AA^, and ECE1c^DD^ in CRC cells. DLD-1 cells overexpressing Flag-tagged ECE1c^WT^, ECE1c^AA^, or ECE1c^DD^ proteins were grown on glass coverslips for 48 h under normal conditions. Cells were observed by confocal microscopy using a specific antibody for Flag and DAPI for nuclei. A representative image is shown from at least ten fields observed for each clone cell. Bar: 20 μm.

Supplementary Table 1 Cell cycle analysis of DLD-1 cells expressing ECE1c^WT^, ECE1c^AA^, or ECE1c^DD^ proteins or mock-transduced cells grown in the absence or presence of 25 μM 5-fluorouracil (5-FU). Cell cycle analysis was performed by flow cytometry. Mean % values are displayed, with an averaged 2.6% SEM.
