# Supplementary figures and images for: Phosphorylation of Endothelin-Converting Enzyme-1c at Serines 18 and 20 by CK2 Promotes Aggressiveness Traits in Colorectal Cancer Cells
Source: Front Oncol. 2020 Jul 30;10:1004. doi: 10.3389/fonc.2020.01004 (PMC7406796; doi:10.3389/fonc.2020.01004)

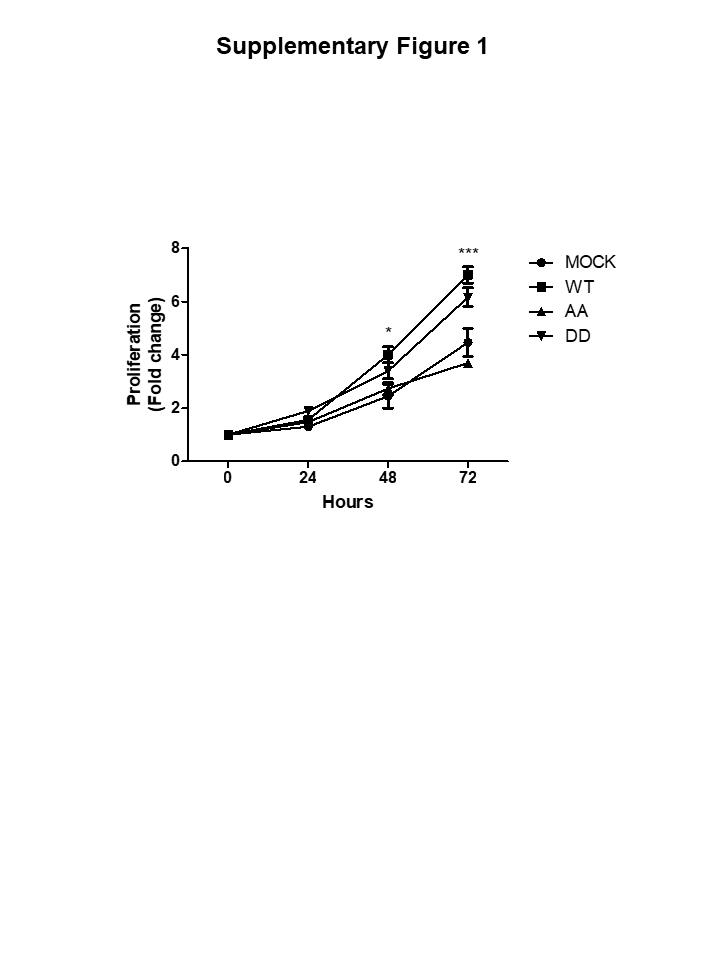

Supplement: Supplementary Figure 1 — ECE1cDD increases proliferation of CRC cells. DLD-1 cells overexpressing Flag-tagged ECE1cWT, ECE1cAA, or ECE1cDD were grown for 24, 48, and 72 h under normal conditions and proliferation was evaluated by Trypan blue staining. Graph represents mean ± SEM; *p < 0.05; ***p < 0.001. [file Image_1.TIF]

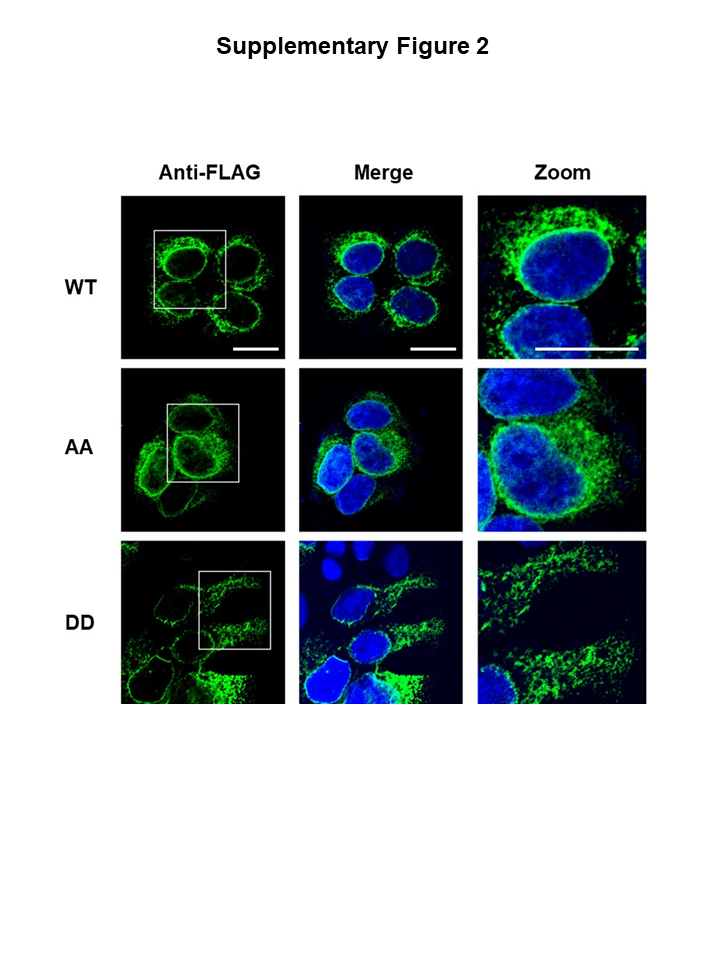

Supplement: Supplementary Figure 2 — Subcellular localization of ECE1cWT, ECE1cAA, and ECE1cDD in CRC cells. DLD-1 cells overexpressing Flag-tagged ECE1cWT, ECE1cAA, or ECE1cDD proteins were grown on glass coverslips for 48 h under normal conditions. Cells were observed by confocal microscopy using a specific antibody for Flag and DAPI for nuclei. A representative image is shown from at least ten fields observed for each clone cell. Bar: 20 μm. [file Image_2.TIF]
