# Supplementary material for: Phosphorylation of Endothelin-Converting Enzyme-1c at Serines 18 and 20 by CK2 Promotes Aggressiveness Traits in Colorectal Cancer Cells
Source: Front Oncol. 2020 Jul 30;10:1004. doi: 10.3389/fonc.2020.01004 (PMC7406796; doi:10.3389/fonc.2020.01004)
Supplement: Supplementary Table 1 — Cell cycle analysis of DLD-1 cells expressing ECE1cWT, ECE1cAA, or ECE1cDD proteins or mock-transduced cells grown in the absence or presence of 25 μM 5-fluorouracil (5-FU). Cell cycle analysis was performed by flow cytometry. Mean % values are displayed, with an averaged 2.6% SEM. [file Table_1.pdf]

Supplementary Table 1

	treatment	$SubG_0$ (%)	G_0/G_1 (%)	S (%)	G_2/M (%)
**Mock**	control	3,1	51,2	12,8	30,0
	5-FU	6,5	68,7	9,1	14,9
**WT**	control	7,0	60,3	11,2	19,7
	5-FU	7,2	69,0	8,8	13,5
**AA**	control	6,3	51,1	18,6	22,2
	5-FU	16,5	53,7	13,0	14,6
**DD**	control	2,9	57,0	14,9	23,6
	5-FU	3,5	68,8	13,8	13,0
